# How to choose the intraocular lens power calculation formulas in eyes with extremely long axial length? A systematic review and meta-analysis

**DOI:** 10.1371/journal.pone.0296771

**Published:** 2024-01-22

**Authors:** Xiaoyu Li, Xiaodong Wang, Xuan Liao

**Affiliations:** 1 Nanchong Central Hospital, The Second Clinical College, North Sichuan Medical College, Nanchong, China; 2 Department of Ophthalmology of Affiliated Hospital, North Sichuan Medical College, Nanchong, China; University of Warmia, POLAND

## Abstract

**Objective:**

To evaluate the accuracy of 10 formulas for calculating intraocular lens (IOL) power in cataract eye with an axial length (AL) of more than 28.0 mm.

**Methods:**

We searched scientific databases including PubMed, EMBASE, Web of Science and Cochrane Library for research published over the past 5 years, up to Sept 2023. The inclusion criteria were case series studies that compared different formulas (Barrett II, EVO, Kane, Hill-RBF, Haigis, Hoffer Q, Holladay 1, SRK/T, Holladay 1 w-k and SRK/T w-k), in patients with extremely long AL undergoing uncomplicated cataract surgery with IOL implantation. The mean difference (MD) of mean absolute error (MAE) and the odds ratio (OR) of both the percentage of eyes within ±0.50D of prediction error (PPE±0.50D) and the percentage of eyes within ±1.00D of prediction error (PPE±1.00D) among different formulas were pooled using meta-analysis.

**Results:**

A total of 11 studies, involving 1376 eyes, were included to evaluate the 10 formulas mentioned above. Among these formulas, Barrett II, EVO, Kane, and Hill-RBF demonstrated significantly lower MAE values compared to SRK/T. Furthermore, Kane and Hill-RBF had lower MAE values than EVO. Additionally, Barrett II and Kane yielded significantly lower MAE values than Haigis while Hill-RBF showed significantly lower MAE values than Holladay 1. Moreover, Hill-RBF showed the highest values for both PPE±0.50D and PPE±1.00D, followed by Kane. Both EVO and Kane had higher values of PPE±0.50D and PPE±1.00D compared to Haigis and SRK/T.

**Conclusion:**

The Wang-Koch adjusted formulas and new-generation formulas have shown potential for higher accuracy in predicting IOL power for cataract patients with extremely long AL compared to traditional formulas. Based on the current limited clinical studies, Hill-RBF and Kane formulas seem to be a better choice for eyes with extremely long AL.

## Introduction

The accuracy of ocular biometric measurements and intraocular lens (IOL) power calculation is essential for achieving satisfactory visual outcomes in cataract patients after surgery [1]. With the advent of optical coherence biometry, the precision of these measurements has significantly improved, surpassing that of traditional ultrasound biometry. Then, the selection of an appropriate IOL calculation formula becomes a critical factor influencing the accuracy of IOL power prediction [2]. Although the IOL power calculation formulas have been greatly improved, the error of the formula is still obvious for dealing with cataract patients with longer axial length (AL) [3]. Finding an appropriate formula is important for cataract patients with extremely long AL, as they often experience hyperopic shifts postoperatively, resulting in reduced satisfaction [4].

In 2018, Melle *et al*. [5] conducted a large-sample study using a single optical biometry device to compare the accuracy of various IOL power calculation formulas in 18501 cataract patients. Their findings revealed that the Barrett II formula demonstrated superior performance compared to other formulas especially in 1548 cataract patients with long AL. However, it is important to note that this study only included patients with an AL less than 28.00 mm. Furthermore, there is still ongoing debate and controversy surrounding the accuracy of IOL calculation formulas for patients with AL more than 28.00 mm.

In recent years, there has been an increase in the utilization of the new-generation formulas, leading to a number of studies exploring their effectiveness. However, previous studies have been limited by the low prevalence and small sample sizes of cataract patients with extremely long AL, leading to a lack of consensus on the conclusions. Thus, we conducted the meta-analysis to provide a comprehensive evaluation of the accuracy of 10 IOL formulas for cataract patients with AL greater than 28.00 mm.

## Methods

The protocol of this meta-analysis was registered prospectively (CRD42023458185) in the PROSPERO database (University of York, United Kingdom). The study adhered to the preferred reporting items for systematic reviews and meta-analysis (PRISMA) statement [6]. The PRISMA Checklist is shown in S1 Table.

### Search strategy

In this meta-analysis, databases including PubMed, EMBASE, Web of Science and Cochrane Library, were searched over the past 5 years up to Sept 2023 using the following search term: (“extremely long axial length*” OR “extremely long eye*” OR “myopi*”) AND (“cataract” OR “IOL” OR “intraocular lens”) AND (“calculat*” OR “formula*”). Two researchers evaluated the title and abstract of all retrieved literature first. After excluding the studies that did not meet the inclusion criteria, the literature was read in full text. Finally, all eligible studies were included.

### Inclusion and exclusion criteria

Articles included in this meta-analysis should meet the following criteria: (I) case series studies; (II) eyes undergoing uncomplicated cataract surgery and IOL implantation; (III) AL longer than 28.00 mm; (IV) use of more than 2 target formulas; (V) all ocular parameters measured by optical biometry. The exclusion criteria were as follows: (I) patients with a history of ophthalmic surgery or ocular trauma, or other eye diseases that may affect vision acuity; (II) mean absolute errors (MAE) or the percentage of eyes within ±0.50D of prediction error (PPE±0.50D) or the percentage of eyes within ±1.00D of prediction error (PPE±1.00D) was not provided; (III) full text not available.

### Data extraction and risk of bias assessment

Data were extracted independently by two researchers (Li X.Y. and Wang X.D.). The third author (Liao X.) made the final decision when a consensus could not be reached. Among the studies of repeated publication, we selected the most informative data with the longest follow-up. The characteristics were extracted from the eligible articles, including author name, publication time, sample size, demographic data (age), AL, IOL type, MAE, PPE±0.50D, PPE±1.00D, follow-up time, and postoperative refraction method. In cases where the MAE and its standard deviation (SD), as well as the values of PPE±0.50D or PPE±1.00D, were not directly provided, calculations were performed based on the original data from the literature.

The quality of evidence was assessed using a modified checklist adapted from the QUADAS-2 tool, which consists of 4 domains: patient selection, index test, reference standard, and flow of patients through the study and timing of the index tests and reference standard (flow and timing). Each domain was evaluated for risk of bias, while the first 3 domains were also assessed for applicability. Risk of bias and applicability are graded as “low risk of bias”, “high risk of bias”, or “unclear” [7]. The funnel plots were used to assess publication bias.

### Statistical analysis

The software program (RevMan, version 5.3) was used to conduct the meta-analysis, and a value of *P*<0.05 was considered statistically significant. We selected MAE, PPE±0.50D and PPE±1.00D as the outcome, and the Mean difference (MD) and odds ratio (OR) and their 95% confidence intervals (CI) of the results were compared. The existence of between-study heterogeneity was investigated through the *I*^*2*^ statistic and the Chi-square test. We used a random-effects model to evaluate pooled effects when *I*^*2*^ values were greater than 50% and *P* < 0.1; otherwise, the fixed-effect model was applied. Finally, sensitivity analysis was conducted to identify possible sources of heterogeneity.

## Results

**Fig 1:**
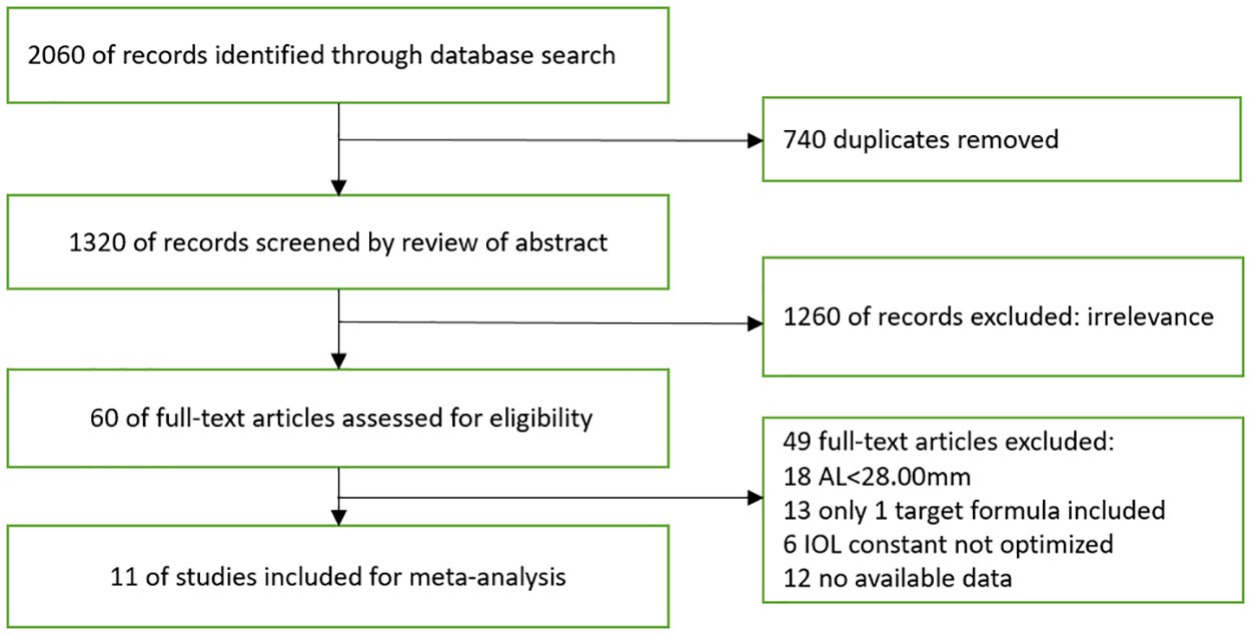
Flowchart of trial selection.

The literature search yielded a total of 2060 potentially relevant articles (Fig 1). After 740 duplicate articles were removed, 1320 remained. After the exclusion of 1260 articles according to the abstract screening, 60 papers remained and underwent full-text assessment. Out of the 60 studies analyzed, 49 studies were excluded for various reasons. Specifically, 18 studies provided AL less than 28.0 mm. Additionally, 13 studies focused on only one target IOL calculation formula. 6 studies did not perform constant optimizations, and 12 studies did not provide the values of MAE, PPE±0.50D and PPE±1.00D. Finally, 11 articles were included in the meta-analysis.

### Characteristics of included studies

Table 1 shows the essential characteristics of the included studies. Among the 11 studies, a total of 1376 eyes were enrolled, of which 1376 eyes were calculated with Barrett II, 1121 eyes with EVO, 1187 eyes with Kane, 1093 eyes with Hill-RBF, 599 eyes with Haigis, 93 eyes with Hoffer Q, 130 eyes with Holladay 1,327 eyes with SRK/T, 289 eyes with Holladay 1 w-k and 289 eyes with SRK/T w-k.

**Table 1:** Characteristics of included studies.

Author/Year	Patients/Eyes	Age (year)	AL (mm)	IOL	Post-op refraction (week)	Refraction method	Formula									
							Barrett II	EVO	Kane	Hill-RBF	Haigis	Hoffer Q	Holladay 1	SRK/T,	Holladay 1 w-k	SRK/T w-k
Bernardes 2021 [8]	NA/41	NA	30.66~35.85	MA60MA	6–8	Subjective	√		√	√	√			√	√	√
Chen 2021 [9]	742/742	60.82±8.92	≥28.00	920HA, 409MP, MC X11 ASP, SN60WF, ZCB00	4	Subjective	√	√	√	√						
Cheng 2020 [10]	213/213	NA	≥28.00	SN60WF, AR40e/E/M, ZA9003, Adapt AO, 920H	4	Subjective	√	√	√	√	√				√	√
Chu 2022 [11]	70/70	55.21±12.02	≥28.00	SA60AT, MA60MA, SN60WF, 920H, 630F, ZCB00, SN60AT	8	Objective	√				√	√	√	√		
Guo 2022 [12]	73/73	58.86±10.95	≥29.00	920H	4–16	NA	√	√	√		√			√		
Ji 2021 [13]	37/37	NA	≥28.00	MC X11 ASP	4	Subjective	√			√	√		√	√		
Mo 2021 [14]	58/58	NA	≥28.00	SN6CWS, SN6AT, 509M	4	Subjective	√	√	√		√			√		
Moshirfar 2022 [15]	25/35	56.94±9.56	≥28.00	MA60MA, AR40e, MX60E, ZCB00, ZCT225, ZXR00	4	Subjective	√	√	√	√					√	√
Omoto 2022 [16]	25/25	NA	≥28.00	ZCB00, ZCB00V	4	Subjective	√		√	√	√			√		
Rong 2019 [17]	59/59	60.76±9.40	≥28.00	920HA	4	Subjective	√				√					
Zhou 2019 [18]	23/23	NA	≥30.00	AcrySof IOL	4	Objective	√				√	√	√	√		

AL, axial length; IOL, intraocular lens; NA, not available

### Risk of bias in individual studies

**Fig 2:**
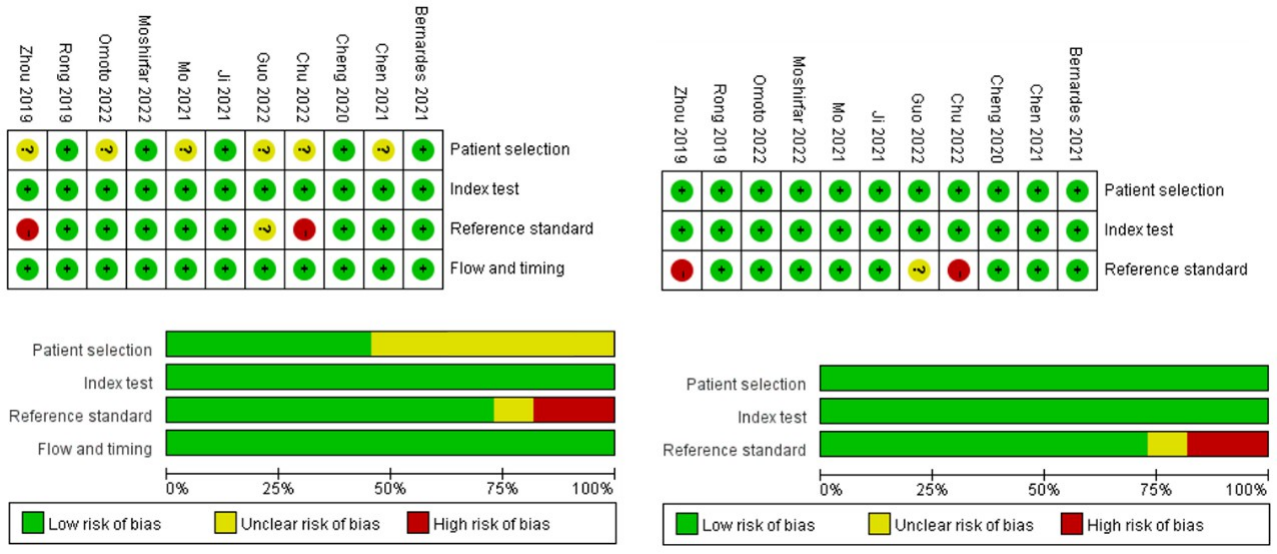
Quality assessment of the eligible studies according to the modified QUADAS-2.

**Fig 3:**
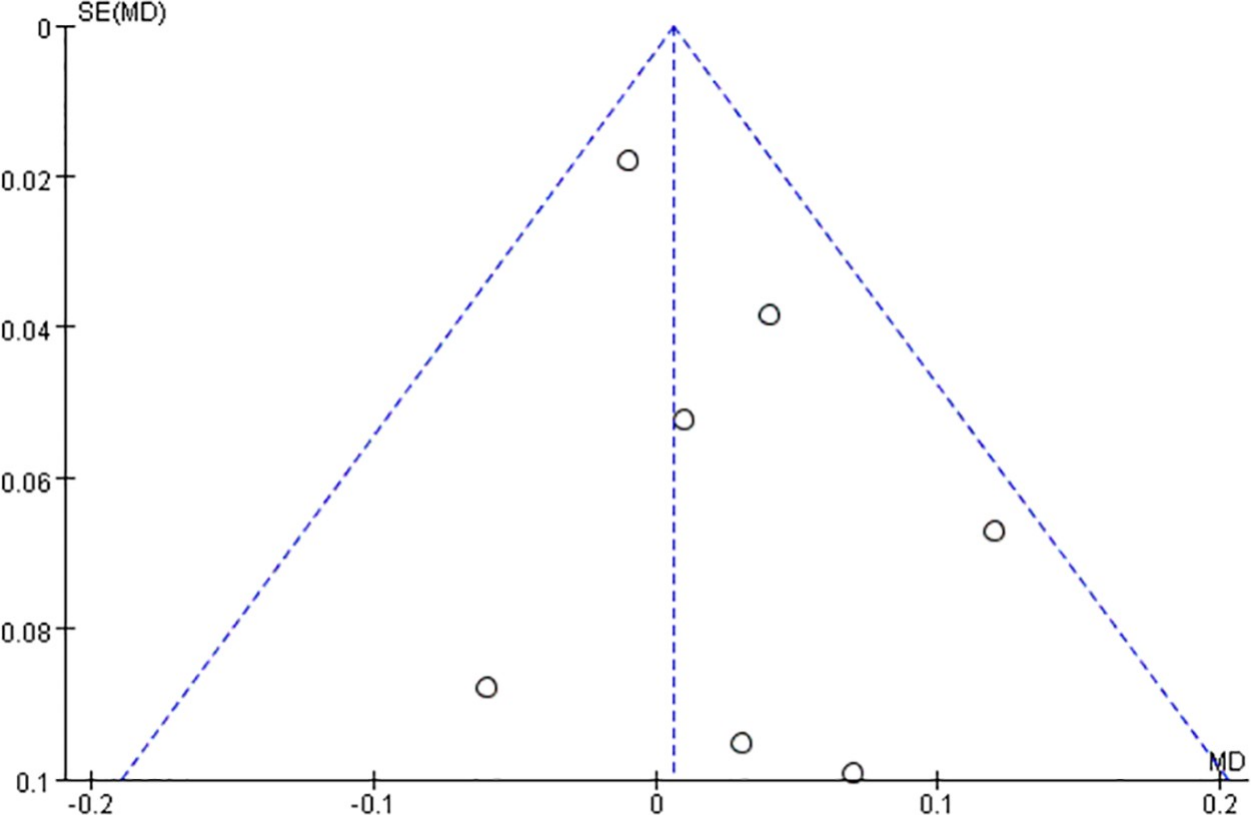
Funnel plot for publication bias test.

The modified QUADAS-2 adapted for risk of bias and applicability was applied to evaluate the quality of the included studies (Fig 2). Of the included articles, six studies did not report whether the enrollment of the patients was consecutive or random, with an unclear risk of bias. For reference standard, most studies performed subjective refraction, two studies with objective refraction, and one study did not state the refraction method; so there was an unknown risk of bias. Most of the literature was of high quality for index test and flow assessment. Finally, we employed a funnel plot to assess the publication bias. As shown in Fig 3, the distribution of the studies was symmetrical on the graph, indicating that the publication bias was negligible.

### Outcomes

**Fig 4:**
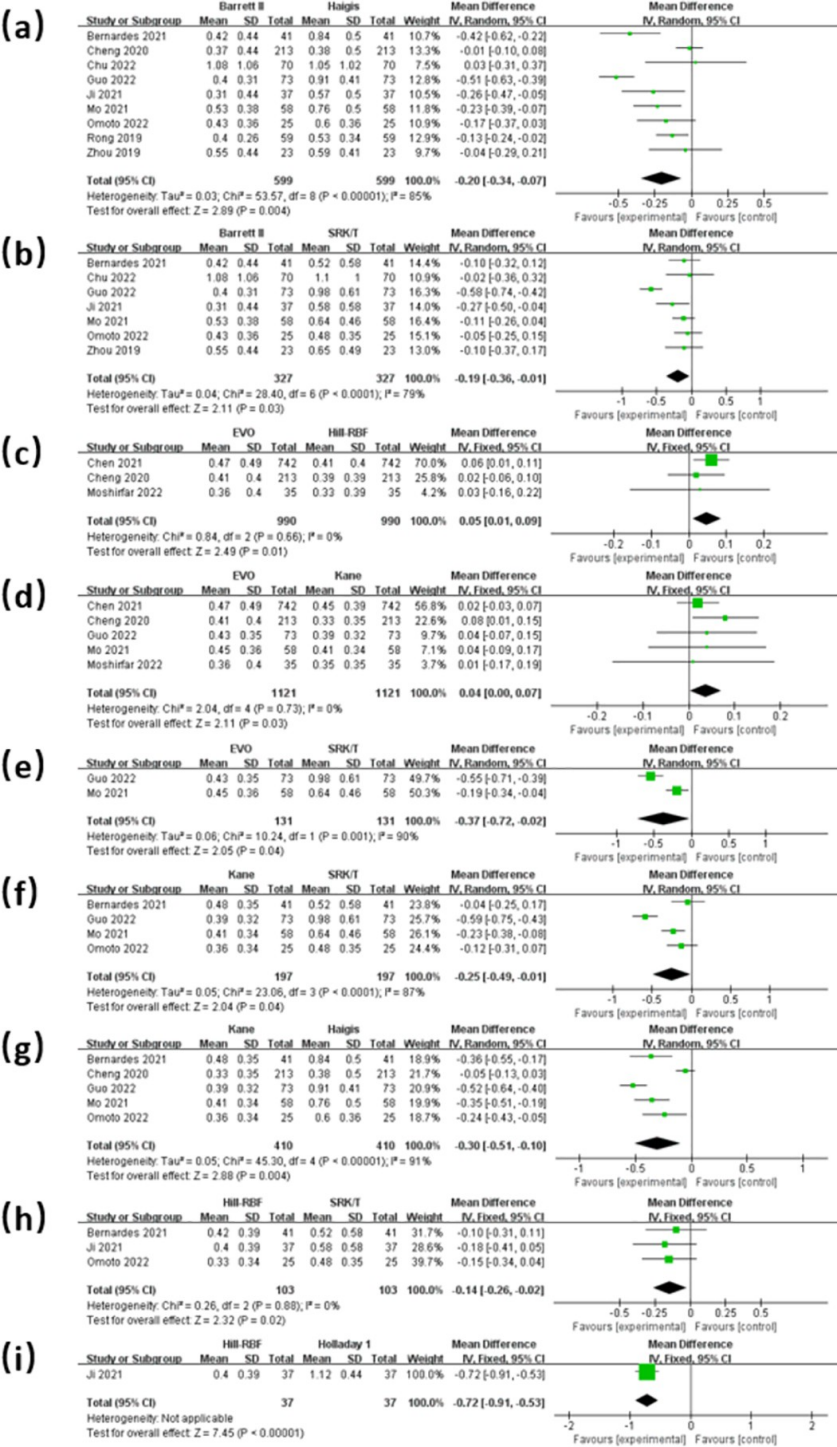
Forest plots comparing the MAE between Barrett II and Haigis (a), Barrett II and SRK/T (b), EVO and Hill-RBF (c), EVO and Kane (d), EVO and SRK/T (e), Kane and SRK/T (f), Kane and Haigis (g), Hill-RBF and SRK/T (h), Hill-RBF and Holladay 1 (i).

Fig 4 shows the forest plots for the comparisons of MAE among different formulas. The MAE of the Barrett II was significantly lower than that of the Haigis (MD = -0.20D, 95% CI: -0.34 to -0.07D, *P* = 0.004) and SRK/T (MD = -0.19D, 95% CI: -0.36 to -0.01D, *P* = 0.03). Kane had a significantly lower MAE than Haigis (MD = -0.30D, 95% CI: -0.51 to -0.10D, *P* = 0.004) and SRK/T (MD = -0.25D, 95% CI: -0.49 to -0.01D, *P* = 0.04). The MAE of the EVO was significantly higher than that of the Kane (MD = 0.04D, 95% CI: 0.00 to 0.07D, *P* = 0.03) and Hill-RBF (MD = 0.05D, 95% CI: 0.01 to 0.09D, *P* = 0.01). Both EVO and Hill-RBF had lower MAE than the SRK/T, and the MAE of Hill-RBF was significantly lower than that of Holladay 1 (MD = -0.72D, 95% CI: -0.91 ~ -0.53D, *P* < 0.001).

**Fig 5:**
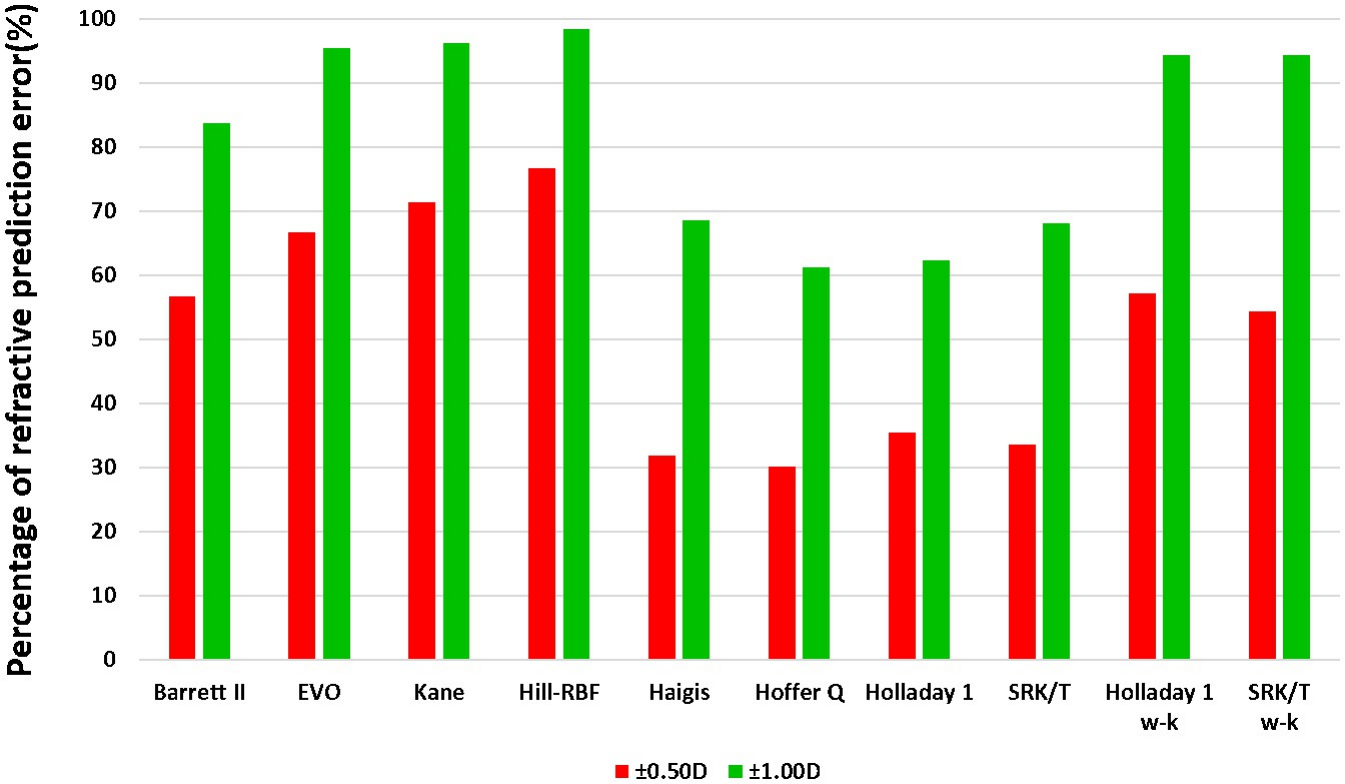
The overall PPE±0.50D and PPE±1.00D of the included formulas.

Fig 5 presents the overall PPE±0.50D and PPE±1.00D values for various formulas, including Barrett II, EVO, Kane, Hill-RBF, Haigis, Hoffer Q, Holladay 1, SRK/T, Holladay 1 w-k, and SRK/T w-k. The percentages for each formula are as follows: 56.64% (83.63%), 66.67% (95.37%), 71.43% (96.24%), 76.67% (98.33%), 31.94% (68.59%), 30.11% (61.29%), 35.48% (62.37%), 33.51% (68.06%), 57.14% (94.29%), and 54.29% (94.29%).

**Fig 6:**
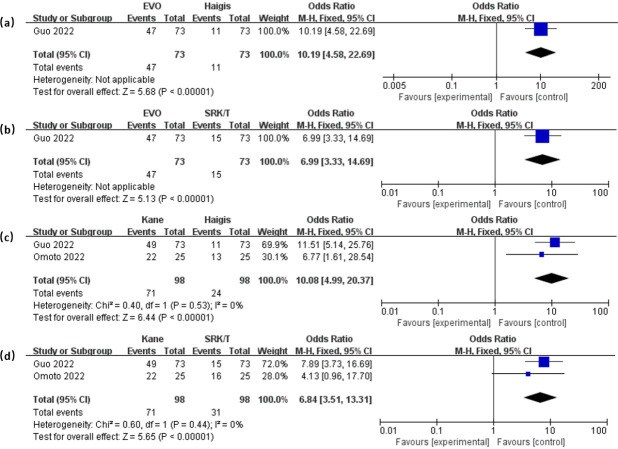
Forest plots of the PPE±0.50D when comparing between EVO and Haigis (a), EVO and SRK/T (b), Kane and Haigis (c), Kane and SRK/T (d).

**Fig 7:**
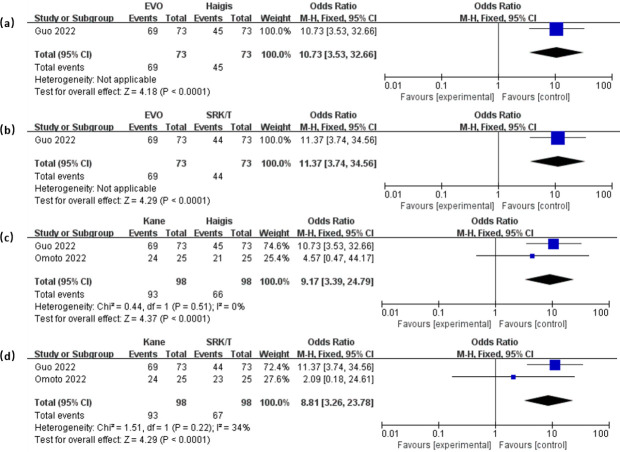
Forest plots of the PPE±1.00D when comparing between EVO and Haigis (a), EVO and SRK/T (b), Kane and Haigis (c), Kane and SRK/T (d).

To illustrate the variance in PPE±0.50D values between different formulas, the forest plots in Fig 6 were used. The EVO showed significantly higher PPE±0.50D compared to Haigis (OR = 10.19, 95% CI: 4.58 to 22.69, *P* < 0.001) and SRK/T (OR = 6.99, 95% CI: 3.33 to 14.69, *P* < 0.001). And Kane exhibited higher PPE±0.50D compared to Haigis (OR = 10.08, 95% CI: 4.99 to 20.37, *P* < 0.001) and SRK/T (OR = 6.84, 95% CI: 3.51 to 13.31, *P* < 0.001). The results for PPE±1.00D, presented in Fig 7, also demonstrated that EVO and Kane outperformed Haigis and SRK/T (all *P* < 0.001). No significant difference was found in the remaining pairwise comparisons. Our full data is available in S2 Table.

### Heterogeneity and sensitivity

Sensitivity analysis showed that by omitting Guo 2022 [12], the *I*^*2*^ value decreased to 0% in the comparison of the MAE between Barrett II and SRK/T, and *I*^*2*^ values had a significantly decreased in the comparison of the MAE between Kane and SRK/T, as well as between Hill-RBF and Haigis. In Guo 2022 [12], patients with a postoperative corrected distant visual acuity (CDVA) of 6/20 or more were included. However, it is important to note that this study deviated from the current guidelines by including patients with CDVA > 20/40, which sets it apart from other studies [19]. This may be the source of heterogeneity, but further verification is needed.

## Discussion

The accuracy of the IOL calculation formula can be determined by PE, which is the difference of the preoperative predicted power using IOL formula minus the postoperative spherical equivalent. Because positive and negative PE can offset each other, its mean absolute value (MAE) is used as an outcome indicator to evaluate the accuracy of the IOL formula. Another commonly used indicator is the PPE±0.50D and PPE±1.00D. A smaller MAE indicates a wider PPE±0.50D and PPE±1.00D range, resulting in better postoperative uncorrected visual acuity and patient satisfaction. In this study, we aimed to evaluate the predicted accuracy of the IOL formula in cataract patients with extremely long AL by calculating the MAE, PPE±0.50D and PPE±1.00D.

To control heterogeneity and biasness, optical biometric instruments were used to measure patient’s eye biological parameters in all included studies. It was observed that optical biometric instruments demonstrated better repeatability and accuracy compared to traditional ultrasound biometric instruments [20]. Furthermore, all the included studies were conducted with optimized IOL constants in order to eliminate systematic errors and minimize the impact of variations in IOL types and patient population characteristics on the calculation formula [21]. Additionally, the postoperative refraction measurements in these studies were performed at a minimum of 4 weeks after surgery. This time frame was chosen based on the general recognition that with small-incision cataract surgery and 1-piece IOLs, the refraction can be considered stable as early as 1 week after surgery, and current guidelines recommend waiting at least 2 weeks to 1 month before performing postoperative refraction measurements [19, 22]. To our knowledge, this is the first meta-analysis that evaluates the accuracy of various IOL power calculation formulas in cataract patients with extremely long AL. The evaluation is based on the calculation of MAE, PPE±0.50D and PPE±1.00D.

The prediction of IOL power calculation was less accurate in patients with long AL, which are commonly defined as AL longer than 24.50 mm [23]. Wang et al. [24] conducted a systematic review and meta-analysis, including 11 observational studies with a total of 4047 eyes. Their findings revealed that the accuracy of Barrett II was superior to SRK/T, Hoffer Q, Holladay 1, and Holladay 2 in cataract patients with long AL. Subgroup analysis further demonstrated that the Barrett II outperformed the Haigis for AL ranging from 24.50 to 26.00 mm, but the difference was not statistically significant when the AL exceeded 26.00 mm. Furthermore, an update meta-analysis research by Li et al. [25] suggested that some new-generation, such as Kane, EVO, LSF, Barrett II, and Hill-RBF, showed promising results in IOL power calculation in cataract patients with long AL, with a higher percentage of eyes having predictable outcomes compared to traditional formulas. Previous studies have consistently shown that as the increase of AL, the deviation of IOL power becomes more significant. In particular, eyes with AL greater than 28.0 mm, which are considered to have extremely long AL, often exhibit over 1.00D difference between predicted refractive error and actual postoperative outcomes [26]. This poses a considerable challenge for cataract surgeons, who currently lack sufficient research on the accuracy of IOL calculation formulas specifically for cataract patients with extremely long AL. Therefore, the present study aimed to address this gap by including cataract patients with extremely long AL, providing valuable evidence for the selection of an appropriate IOL formula in such cases.

Our results revealed significant differences in the MAE values among different IOL power calculation formulas. Specifically, Barrett II, EVO, Kane and Hill-RBF exhibited lower MAE values compared to SRK/T. Barrett II and Kane had lower MAE values than Haigis but Hill-RBF outperformed Holladay 1. These results indicated that new-generation formulas exhibit a promising potential for higher accuracy in predicting IOL power for cataract patients with extremely long AL, as compared to traditional formulas. In addition, PPE±0.50D and PPE±1.00D values are clinically important as they may affect patients’ postoperative satisfaction. Previously, Gale et al. [27] established a benchmark standard for the UK National Health Service in 2009 by utilizing partial coherence interferometry biometer and incorporating optimization constants for IOL calculation. Originally, the benchmark standard required patients to achieve PPE±0.50D values of 55% and PPE±1.00D values of 85%. This standard was revised and increased in 2019, with the updated target being 62.36% for PPE±0.50D and 88.76% for PPE±1.00D [28]. According to our study findings, the PPE±0.50D values of the new-generation formulas (Barrett II, EVO, Kane and Hill-RBF) and Wang-Koch adjusted formulas (Holladay 1 w-k and SRK/T w-k) ranged from 54.29% to 76.67%. Additionally, the PPE±1.00D values ranged from 83.63% to 98.3%. These results indicated that these formulas almost meet the desired benchmark standards. Notably, Hill-RBF demonstrated outstanding performance by exhibiting the highest values for both PPE±0.50D and PPE±1.00D, with the Kane ranking second in terms of performance. Conversely, the traditional formulas (Haigis, Hoffer Q, Holladay 1, and SRK/T) exhibited PPE±0.50D values ranging from 30.11% to 35.48% and PPE±1.00D values ranging from 61.29% to 68.59%, all of which were below the recommended benchmark standards. It is important to note that only EVO and Kane demonstrated significantly higher values of PPE±0.50D and PPE±1.00D when compared to Haigis and SRK/T, with no statistically significant differences observed in the remaining comparisons. These nonsignificant findings may be attributed to the small sample sizes and limited statistical power resulting from some comparisons based on single relevant studies.

Consistent with previous findings, this study demonstrated that traditional formulas could produce significant errors in patients with extremely long AL [12]. The Hoffer Q, Holladay 1 and SRK/T formulas used parameters only AL and keratometry (K) but not preoperative anterior chamber depth (ACD), which reduces their accuracy in predicting the effective lens position (ELP). The Haigis formula incorporates the function ELP = a0 + (a1 × ACD) + (a2 × AL) to derive ELP. However, being a thin-lens formula, it is greatly influenced by the change of lens thickness (LT), which may lead to potential prediction inaccuracies [29]. Among these formulas, the SRK/T formula has been recommended for patients with long AL [30]. However, its accuracy may be compromised for patients with extremely long AL cataracts. This discrepancy could be attributed to the assumption made by the SRK/T formula that longer eyes would have deeper ACD. In reality, the association between AL and ACD may not be as straightforward as assumed. Furthermore, the SRK/T formula utilizes the Fyodorov method to calculate ELP, and due to the lack of strict boundary values, it is highly sensitive to changes in K values. Clinical studies have consistently demonstrated that the accuracy of the SRK/T formula is particularly influenced by variations in K values compared to other formulas [5]. In theory, a larger eyeball and longer AL are associated with a flatter corneal curvature [31]. Consequently, patients with an extremely long AL who have smaller K values may experience reduced accuracy when using the SRK/T formula. Additionally, the SRK/T formula exhibits non-physiological behavior, which further contributes to errors in IOL power prediction for patients with extremely long AL [32].

The Wang-Koch adjusted formulas and new-generation formulas have shown high predictive accuracy in patients with extremely long AL. Among these patients, the use of a single average value for whole-eye refractive index leads to overestimation of AL, so they tend to choose IOLs with insufficient power and result in hyperopic accidents. This is a problem with the traditional formulas. To address this issue, Wang et al. [33] proposed a method of optimizing the axial length, thereby enhancing the accuracy of different traditional formulas (Wang-Koch adjusted formulas). By employing this optimized AL approach, we can effectively reduce the measurements obtained through optical biometric instruments, thus mitigating the problem of hyperopia accidents caused by traditional formulas in cases involving extremely long AL. The new-generation formula Barrett II is a ray-tracing thick-lens formula. Unlike the traditional formulas, the Barrett II takes into account the varying principal planes among different power IOLs. Its calculation includes mandatory parameters such as AL, K, ACD, and optional parameters like LT and white to white (WTW). The EVO formula, based on emmetropization theory, is a thick lens formula that accurately calculates IOLs of various geometry and powers. Like the previous formulas, takes into account mandatory variables like AL, K, ACD, and optional variables such as LT and CCT for calculating IOL refractive power [34]. The Kane formula incorporates regression and artificial intelligence components to refine its predictions. It is based on theoretical optics and has a database containing the postoperative refractive status of approximately 30 000 cataract patients. This formula utilizes efficient cloud-based algorithms and considers several variables including mandatory ones like AL, K, ACD and patient gender. Optional variables such as LT and CCT further enhance its predictive capabilities [35]. Notably, the Hill-RBF formula stands out as the most accurate formula for eyes with extremely long AL in this study. It uses pattern recognition, data interpolation, and artificial intelligence to predict postoperative refraction. The formula takes into account 7 variables, including 3 mandatory ones (AL, K, ACD) and 4 optional ones (LT, CCT, WTW and patient gender), for calculating IOL refractive power. Its promising accuracy may be attributed to its unique calculation pattern and the fact that its methodology is data-driven and free of calculation bias. As more data are accumulated in the dataset, it is expected that the accuracy of this method will be further improved, enabling the calculation of powers for patients with extremely long AL [36].

The present meta-analysis has several limitations and needs to be further investigated. First, the number of cases included in this study was limited, due to the low proportion of cataract patients with AL ≥ 28.00 mm. Second, there were few studies available on formulas such as Hoffer Q, Holladay 1, SRK/T, Holladay 1 w-k and SRK/T w-k, and some comparisons were based on only one relevant study. Therefore, this leaded to small sample sizes and insufficient power when compared to other formulas. Third, the impact of patient population characteristics and IOL types on the predicted accuracy of the IOL formula were not considered. Additionally, our study did not include newer IOL formulas like Olsen, Ladas Super, Panacea, and Pearl-DGS due to the short application time and limited availability of relevant studies.

In conclusion, the Wang-Koch adjusted formulas and new-generation formulas have shown potential for higher accuracy in predicting IOL power for cataract patients with extremely long AL compared to traditional formulas. Among these formulas, Hill-RBF and Kane formulas appear to have a superior performance in calculating IOL power for eyes with extremely long AL. However, it is important to acknowledge that the existing clinical research is currently limited. Therefore, additional follow-up studies with sufficient randomized controlled trial data are necessary to confirm the accuracy of these formulas specifically for eyes with extremely long AL.

## Supporting information

S1 Table PRISMA 2020 checklist. (DOCX)

S2 Table Data. (XLSX)
