# Correction: How to choose the intraocular lens power calculation formulas in eyes with extremely long axial length? A systematic review and meta-analysis

**DOI:** 10.1371/journal.pone.0325030

**Published:** 2025-05-21

**Authors:** Xiaoyu Li, Xiaodong Wang, Xuan Liao

In the Funding statement, the grant number from the funder Project of the National Natural Science Foundation of Sichuan Provincial Department of Science and Technology is listed incorrectly. The correct grant number is: 2023NSFSC0595.
